# Platelet-derived HMGB1 induces NETosis, exacerbating brain damage in the photothrombotic stroke model

**DOI:** 10.1186/s10020-025-01107-7

**Published:** 2025-02-05

**Authors:** Sang-A. Oh, Song-I. Seol, Dashdulam Davaanyam, Seung-Woo Kim, Ja-Kyeong Lee

**Affiliations:** 1 Department of Anatomy, Inha University School of Medicine, Iinha 100, Nam-Gu, Inchon, 22212 Republic of Korea; 2 Department of Biomedical Sciences, Inha University School of Medicine, Inchon, Republic of Korea

**Keywords:** HMGB1, NETosis, Photothrombosis, Platelet, TLR4

## Abstract

Following cerebral ischemia, neutrophil extracellular traps (NETs) contribute significantly to brain damage by exacerbating delayed immune cell infiltration and vascular injury. They are detected both in brain tissue and within blood vessels. Danger-associated molecular pattern (DAMP) molecules have been implicated in inducing NETosis after cerebral ischemia. This study investigated the role of High mobility group box 1 (HMGB1), a prototype DAMP molecule, in NETosis induction following photothrombotic stroke (PTS), with a particular focus on neutrophil-platelet interactions. In PTS, thrombi consist primarily of aggregated platelets and neutrophils, lacking significant fibrin content. Triphenyltetrazolium chloride (TTC) staining revealed rapid but progressive expansion of the infarct area in the PTS model, commencing within 1 h and continuing until 24 h. Concomitant with this, peripheral neutrophils isolated following PTS exhibited progressive NETosis, particularly intravascular NETosis. This was evidenced by significant increase in citrullinated histone H3 (CitH3), a marker of NETosis, as early as 1 h post-PTS. Furthermore, serum levels of free DNA gradually and significantly increased, further supporting the induction of NETosis following PTS. Intranasal administration of BBCA, a peptidylarginine deiminase (PAD) inhibitor, effectively suppressed the induction of intravascular NETosis. Importantly, BBCA administration, both 30 min before and 4 h after PTS surgery, significantly reduced infarct volumes at 24 h and improved neurological outcomes. These findings underscore the crucial role of NETosis in both the initiation and progression of ischemic brain damage in this model. Following PTS, HMGB1 rapidly accumulated in serum, detectable as early as 1 h. Immunofluorescence staining revealed initial localization of HMGB1 in neurons, followed by its accumulation within activated neutrophils and platelets within blood vessels. Functional inhibition of HMGB1 by intranasal administration of an HMGB1 A box 4 h post-PTS significantly suppressed NETosis induction, reduced infarct volume, and improved neurological deficits, confirming the pivotal role of HMGB1 in NETosis induction. Notably, we observed a rapid platelet activation and concomitant HMGB1 induction within activated platelets after PTS. Co-culture experiments using naïve PMNs-platelets isolated following PTS demonstrate that extracellular HMGB1, particularly one derived from platelets, plays a critical role in activating neutrophils and inducing intravascular NETosis via a TLR4-dependent manner. Collectively, these findings highlight the critical role of NETosis not only in the initial stages of thrombus formation but also in the subsequent progression of ischemic brain damage in the PTS animal model. HMGB1, particularly platelet-derived HMGB1, emerges as a key mediator to this process. Therefore, targeting NETosis through modulation of HMGB1 presents a promising multipotent therapeutic strategy for mitigating ischemic brain damage.

**Supplementary Information:**

The online version contains supplementary material available at 10.1186/s10020-025-01107-7.

## Introduction

Neutrophils, the most abundant white blood cells, are rapidly recruited to infection sites to combat pathogens via various means, including production of proinflammatory cytokines, reactive oxygen species (ROS), and antibacterial peptides (Amantea et al. 2009). Recently, neutrophil extracellular traps (NETs), formed by the release of decondensed chromatin and granular contents, have been reported as a powerful antimicrobial defense mechanism (Brinkmann et al. 2004; Papayannopoulos et al. 2010). While NETs are crucial for fighting infections, extensive NET formation has been linked to various non-infectious inflammatory conditions and the perpetuation of inflammation and tissue damage (Fuchs et al. 2007; Kaplan and Radic 2012). For instance, extensive NET formation is believed to aggravate autoimmune diseases, such as RA (Spengler et al. 2015), systemic lupus erythematosus and lupus nephritis (Villanueva et al. 2011; Mistry and Kaplan 2017) and contribute to sterile inflammatory conditions such as atherosclerosis, venous thrombosis, lung injury, and tumor metastasis (Caudrillier et al. 2012; Franck et al. 2018; Kimball et al. 2016; Tohme et al. 2016).

In the central nervous system, neutrophils rapidly migrate to and accumulate at sites of brain injury under various pathological conditions. NETosis induction has been observed not only in brain tissues but also within blood vessels in diverse animal models of stroke (Perez-de-Puig et al. 2015; Kim et al. 2019; Kang et al. 2020) and in the thrombus or plasma of patients experiencing acute ischemic stroke (Laridan et al. 2017; Valles et al. 2017; Ducroux et al. 2018). Our recent work demonstrated that NETosis induction exacerbates neuronal damage in the post-ischemic brain, with high mobility group box 1 (HMGB1) and ATP, both recognized as danger-associated molecular patterns (DAMPs), playing critical roles (Kim et al. 2019, 2020). Furthermore, NET formation has been implicated in a mouse model of Alzheimer’s disease (Zenaro et al. 2015) and elevated levels of the MPO-DNA complex, a hallmark of NETosis, have been detected in the serum of individuals with multiple sclerosis (Naegele et al. 2012).

HMGB1 has been implicated as a potent inducer of NETosis in various non-infectious diseases. In a liver ischemia/reperfusion injury model, treatment with recombinant HMGB1 upregulated CitH3 expression and stimulated NET formation, and these effects were mediated through Toll-like receptor (TLR)4/9 signaling (Huang et al. 2015). Furthermore, HMGB1 triggered the formation of prothrombotic NETs in an animal model of deep venous thrombosis, a process mediated by the receptor for advanced glycation end products (RAGE) (Stark et al. 2016). Our previous research demonstrated that HMGB1 induces NETosis in a stroke model utilizing permanent middle cerebral artery occlusion (MCAO) induced by the suture method (Kim et al. 2019). We further showed that HMGB1 released by NETting neutrophils establishes a vicious cycle that amplifies the inflammatory response during permanent MCAO (Kim et al. 2019). Recently, Denorme et al. (2022) reported elevated HMGB1 levels in both the plasma and platelets of stroke patients. This finding suggests that platelets may serve as a crucial source of HMGB1, significantly contributing to NET formation during the acute phase of stroke.

Among various stroke models, the PTS model offers several advantages. This model employs the photoactive dye, Rose Bengal (RB), which generates ROS upon illumination. These ROS cause endothelial damage and platelet activation, ultimately leading to thrombus formation (Watson et al. 1985; Dietrich et al. 1986; Uzdensky 2018). PTS model generates a well-defined location and size of the ischemic lesion, low invasiveness and surgical intervention, and prolonged sensorimotor impairment, enabling long-term study of behavioral impairment and recovery (Uzdensky 2018). In the PTS model, thrombi primarily consist of neutrophils and platelets, making it an ideal model for investigating the interplay between these cell types. In the present study, we used a rat model of PTS to examine the temporal progression of intravascular NETosis and investigate its contribution to ischemic brain damage. Additionally, we explored the role of HMGB1 in NETosis induction, specifically focusing on the contribution of platelet-derived HMGB1 to neutrophil activation.

## Materials and methods

### RB photothrombotic rat model

Male Sprague–Dawley rats aged 7–8 weeks were housed under conditions with diurnal lighting and had access to food and tap water ad libitum. All animal procedures adhered strictly to the recommendations outlined in the Guide for the Care and Use of Laboratory Animals published by the National Institutes of Health (NIH, USA, 2013) and followed the ARRIVE guidelines (http://www.nc3rs.org/ARRIVE, accessed on August 31, 2021). The animal protocol underwent ethical review and was approved by the INHA University Institutional Animal Care and Use Committee (INHA-IACUC) prior to implementation (approval number INHA180105-531-2). Surgical procedures for the PTS animal model were performed according to Schmidt et al. (2012) with some modification. Briefly, 8-week-old male Sprague–Dawley rats (250–300 g) were anesthetized with an intramuscular injection of a ketamine (3.75 mg/100 g body weight) and xylazine hydrochloride (0.5 mg/100 g body weight) mixture. Tail veins were dilated with warm water at 37 °C, and then RB (3 mg/kg) was injected into the tail vein using a 26 G syringe. After allowing 5 min for RB to circulate to the brain, the rats were positioned in a stereotactic frame, and the scalp was longitudinally incised and retracted to expose the skull. Two laser beams, each with a diameter of 5 mm and a wavelength of 100 W, were stereotactically positioned onto the skull—one 0.5 mm anterior to the bregma and 3.5 mm lateral from the midline, and the other at a corresponding position on the opposite side. The skull was illuminated for 20 min, and the incision site was sutured. The control group received only an infusion of RB without light exposure. A laser Doppler flowmeter (Periflux System 5000; Perimed, Jarfalla, Sweden) was used to monitor regional cerebral blood flow (CBF) and relative CBF, and a thermoregulated heating pad and a heating lamp were used to maintain a rectal temperature of 37.0 ± 0.5 °C.

### Infarct volume assessment

Rats were sacrificed, and their whole brains were dissected coronally into 2-mm slices using a metallic brain matrix (RBM-40000, ASI, Springville, UT, USA). The slices were immediately incubated in saline containing 2,3,5-triphenyl tetrazolium chloride (TTC 2%) at 37 °C for 15 min and then fixed in 4% paraformaldehyde (PFA, FUJIFILM Wako pure Chemical, Osaka, Japan). Scion Image (Scion Corporation, Frederick, MD, USA) was used to measure infarcted tissue areas. To adjust for edema and shrinkage, the areas of ischemic lesions were calculated using the formula: contralateral hemisphere volume—(ipsilateral hemisphere volume—measured injury volume). The infarct volumes were quantified (mm^3^) by summing the infarct sizes in adjacent tissue sections.

### Modified Neurological Severity Score (mNSS)

Neurological deficits were assessed using the mNSS at 1-day post-PTS. The mNSS evaluates motor, sensory, balance, and reflex functions, with scores ranging from 0 to 18 (0 indicates normal function and 18 indicates maximal deficit) (Chen et al. 2001). Motor function was evaluated using two tests: (1) suspending the rat by its tail and scoring forelimb flexion, hindlimb flexion, and head movement > 10° with respect to the vertical axis on a scale from 0 to 1 (total score 0–3) within 30 s; and (2) placing the rat on the floor and scoring walking ability from 0 to 3 (0 for normal walking, 1 for inability to walk straight, 2 for circling toward the paretic side, and 3 for falling on the paretic side). Sensory function was tested using a placement test and a proprioceptive test, each scored from 0 to 1. Balance was assessed using the beam balance test, with scores ranging from 0 to 6 based on performance: 0 for maintaining a steady posture, 1 for grasping the side of the beam, 2 for hugging the beam with one limb off, 3 for hugging the beam with two limbs off or rotating around the beam for over 60 s, 4 for falling off the beam within 20–40 s, 5 for falling off within 20 s, and 6 for making no attempt to balance or hang onto the beam. Reflexes were scored on four items (maximum possible score of 4): pinna reflex (0–1); corneal reflex (0–1); startle reflex (0–1); and the presence of seizures, myoclonus, or myodystony (0–1).

### Drug administration

Drugs were administered intranasally, as previously described (Kim et al. 2018). Rats were anesthetized via intramuscular injection of a mixture of ketamine (3.75 mg/100 g body weight) and xylazine hydrochloride (0.5 mg/100 g body weight). Following anesthesia, a nose drop containing HMGB1 A box (5 μg/kg; HM-012, HMGbiotech, Milano, Italy) dissolved in DW (100 µL) or BB-Cl-amidine (BBCA, 10 µg/kg; HY-111347A, MedchemExpress, NJ, USA) dissolved in DW (1 mL) was then carefully placed in each nostril of the anesthetized animals (positioned supine at a 90° angle) using a preautoclaved pipet tip (T-200-Y; Axygen, Union, CA, USA). The administration procedure was repeated until the entire dose was administered, with 2-min intervals between applications.

### Serum and brain tissue sample preparation

The frontal cortices and striata of rat brains were isolated and frozen in liquid nitrogen for subsequent experiments after anesthetizing the mice with an intramuscular injection of a mixture containing ketamine (100 mg/kg body weight) and xylazine hydrochloride (23.32 mg/kg body weight). For serum samples, blood was collected through cardiac puncture using a 23 G syringe and left at room temperature for 30 min. Afterward, the blood samples were centrifuged at 6000×*g* and 4 °C for 10 min. The supernatant was then aliquoted and stored at − 80 °C.

### Isolation of circulating neutrophils

Circulating neutrophils were isolated from the collected blood samples using gradient density centrifugation with Histopaque solution (Sigma-Aldrich, St. Louis, MO, USA), following a previously described protocol (Kim et al. 2019). Initially, Histopaque 1077 (3 mL) was layered on Histopaque 1119 (3 mL) in a 15 mL polypropylene tube and collected blood (3 mL) was carefully added on top of this mixture (Histopaque 1077/1119). The layered system, composed of three components, was centrifuged at 400×*g* for 30 min at room temperature. After centrifugation, the first ring of cells (mononuclear cells) was discarded, and the second ring (neutrophils) was transferred to a fresh 15 mL polypropylene tube containing phosphate-buffered solution (PBS) with 0.1% bovine serum albumin and 10% glucose (PBS-BG). The suspension was centrifuged at 1500 × g for 10 min at room temperature. To eliminate residual red blood cells, the resulting pellet was resuspended in 3 mL PBS-BG, poured onto Histopaque-1119 (3 mL), and centrifuged at 1500×*g* for 10 min at room temperature. The neutrophil ring was then transferred to a fresh 15 mL polypropylene tube containing PBS-BG and centrifuged at 1500×*g* for 10 min at room temperature. Finally, the pellet was resuspended in RPMI (Gibco BRL, Gaithersburg, MD, USA) containing 1% FBS.

### Isolation of platelets from rat blood samples

After blood was drawn, it was placed into a Becton Dickinson Vacutainer^®^ (Becton, Dickinson and Company, Franklin Lakes, NJ, USA) containing Acid Cutrate Dextrose with a yellow cap. The samples were gently mixed after blood collection by slowly inverting the Vacutainer^®^. The blood was then centrifuged at 200×*g* for 20 min to obtain platelet-rich plasma (PRP), which was subsequently centrifuged at 100×*g* for 10 min in HEPES buffer [140 mM NaCl, 2.7 mM KCl, 3.8 mM HEPES, 5 mM EGTA (pH 7.4)] in the presence of 1 μM prostaglandin E1 (PGE1; Sigma-Aldrich, St. Louis, MO, USA). Platelets were pelleted by centrifuging the supernatant at 800×*g* for 10 min at room temperature. The platelets were then washed three times with platelet wash buffer [10 mM sodium citrate, 150 mM NaCl, 1 mM EDTA, 1% (w/v) dextrose (pH 7.4)] in the presence of 1 μM PGE1. Finally, the resulting pellet was resuspended in RPMI medium (Gibco BRL) containing 1% FBS.

### Platelet-induced NET formation in co-culture assay

Neutrophils were isolated from freshly collected whole blood and seeded onto 6-well plates (SPL Life Sciences, Gyeonggi, South Korea) at a density of 1 × 10^6^ cells/mL. Activated platelets, generated by PTS, were adjusted to 1 × 10^8^ cells/mL in RPMI (Gibco BRL) containing 1% FBS and placed into the insert. Neutrophils and activated platelets were co-cultured at a 1:100 ratio to induce NETs for 2.5 h at 37 °C in 5% CO_2_/95% air. Adenosine triphosphate (ATP; Sigma-Aldrich, St. Louis, MO, USA) was used as a positive control. PMNs were pre-treated with HMGB1 A box (100 ng/mL; HM-012, HMGbiotech) or A438079 (20 µM, Tocris Bioscience, Bristol, Avon, UK) for 30 min and then co-cultured with platelets. For signaling pathway experiments, PMNs were pre-treated with TLR4 antagonist (TLR4-IN-C34, 20 µM, Sigma-Aldrich), C-X-C chemokine receptor type 4 (CXCR4) receptor antagonist (AMD3100, 20 µg/mL, Sigma-Aldrich), and RAGE antagonist (FPS-ZM1, 150 nM, Sigma-Aldrich) and then co-cultured with platelets.

### Immunoblotting

Brain homogenates were extracted using RIPA buffer [50 mM Tris–HCl (pH 7.4), 150 mM NaCl, 1 mM EDTA, 0.5% NP40, 0.25% sodium-deoxycholate, 0.5% Triton X-100, 10% glycerol, and Complete Mini Protease Inhibitor Cocktail tablet (Roche Diagnostics, Basel, Switzerland)]. Cell or tissue extracts were then loaded on 9–13% Sodium Dodecyl Sulphate–Polyacrylamide Gel Electrophoresis gels and immunoblotted using the following primary antibodies: anti-HMGB1 (1:2000, ab18256, Abcam, Cambridge, UK), anti-citH3 (1:1000, ab5103, Abcam), anti-GAPDH (1:10,000, 14C10, Cell Signaling Technology, Danvers, MA, USA), anti-MPO (1:1000, PA5-16672, Invitrogen, Waltham, MA, USA), anti-CD42b (1:1000, 12860-1-AP, Proteintech, IL, USA) and anti-CD62P (1:200, sc8419, Santacruz, CA, USA). The blots were detected using anti-rabbit HP conjugated or anti-mouse HP secondary antibodies (1:4000, Merck Millipore, Burlington, MA, USA) and a chemiluminescence kit (Merck Millipore).

### Immunofluorescence staining

Brains were fixed in 4% PFA solution for 2 days at 4 °C, post-fixed in a 30% sucrose solution at 4 °C, sectioned at 40 μm using a vibratome, and immunologically stained. Sections were then blocked with 5% FBS, 5% horse serum, and 2% albumin in 0.1% Triton X-100 for 1 h at room temperature. Primary antibodies aganist mouse anti-laminin (NBP2-42392; Novus Biologicals, Centennial, CO, USA), rat anti-Ly6g (ab25024; Abcam, Cambridge, UK), rabbit anti-citH3 (ab5103, Abcam), rabbit anti-HMGB1 (ab18256, Abcam), mouse anti-HMGB1 (ab190377; Abcam, Cambridge, UK) and anti-CD42b (12860-1-AP; Proteintech) were diluted to 1:200. After incubation with primary antibodies, the brain sections were washed with PBS and incubated with rhodamine-labeled anti-rabbit IgG for anti-citH3, FITC-labeled anti-rat IgG for anti-Ly6g, FITC-labeled anti-rabbit IgG for anti-HMGB1, and FITC-labeled anti-mouse IgG for anti-HMGB1 in PBS for 1 h. All antibodies were diluted 1:200 and purchased from Merck Millipore Corporation. The sections were mounted on slides with VECTASHIELD Antifade Mounting Solution containing DAPI (Vector Laboratories) and examined under a Zeiss LSM 510 META microscope (Carl Zeiss Meditec AG, Jena, Germany).

### Immunofluorescence staining of platelets

Isolated platelets were cytocentrifuged onto glass slides at 800 rpm for 5 min at room temperature using a Cytospin system (ThermoFisher Scientific, Waltham, MA, USA). The platelets were fixed with 4% PFA for 20 min at room temperature, followed by overnight incubation in 1% PFA at 4 °C for enhanced fixation. The cytospin slides were blocked with 1% normal goat serum for 1 h at room temperature to reduce nonspecific binding. Subsequently, the slides were incubated with an anti-HMGB1 antibody overnight at 4 °C. Images were acquired using a Zeiss LSM 510 META confocal microscope (Carl Zeiss Meditec AG).

### Enzyme-linked immunosorbent assay (ELISA)

Serum HMGB1 levels were assessed using ELISA kits (Cusabio, Houston, TX, USA), according to the manufacturer's instructions. For serum samples, whole blood was collected at 1, 3, 6, 12 and 24 h after PTS, and allowed to clot by leaving it undisturbed for 30 min at room temperature. The clots were then removed by centrifuging the samples at 6000 rpm for 10 min in a cold microcentrifuge. The supernatants were immediately transferred to clean polypropylene tubes, and their concentrations were determined using ELISA kits.

### Quantification of serum DNA

The serum DNA content was quantified using a Quant-iT PicoGreen double-stranded DNA (dsDNA) assay kit (Invitrogen, Carlsbad, CA, USA) according to the manufacturer’s instructions, and fluorescence was measured using a Gen5 microplate reader (BioTek, Winooski, VT, USA) at an excitation/emission wavelength of 504/523 nm.

### Statistical analysis

Statistical analysis was performed using PRISM software 5.0 (Graph Pad Software). ANOVA was initially used, followed by the Newman–Keuls test. The data are presented as means ± SEMs, with the p-values < 0.05 considered statistically significant.

## Results

### Rapid but progressive infarct expansion in PTS model

**Fig. 1:**
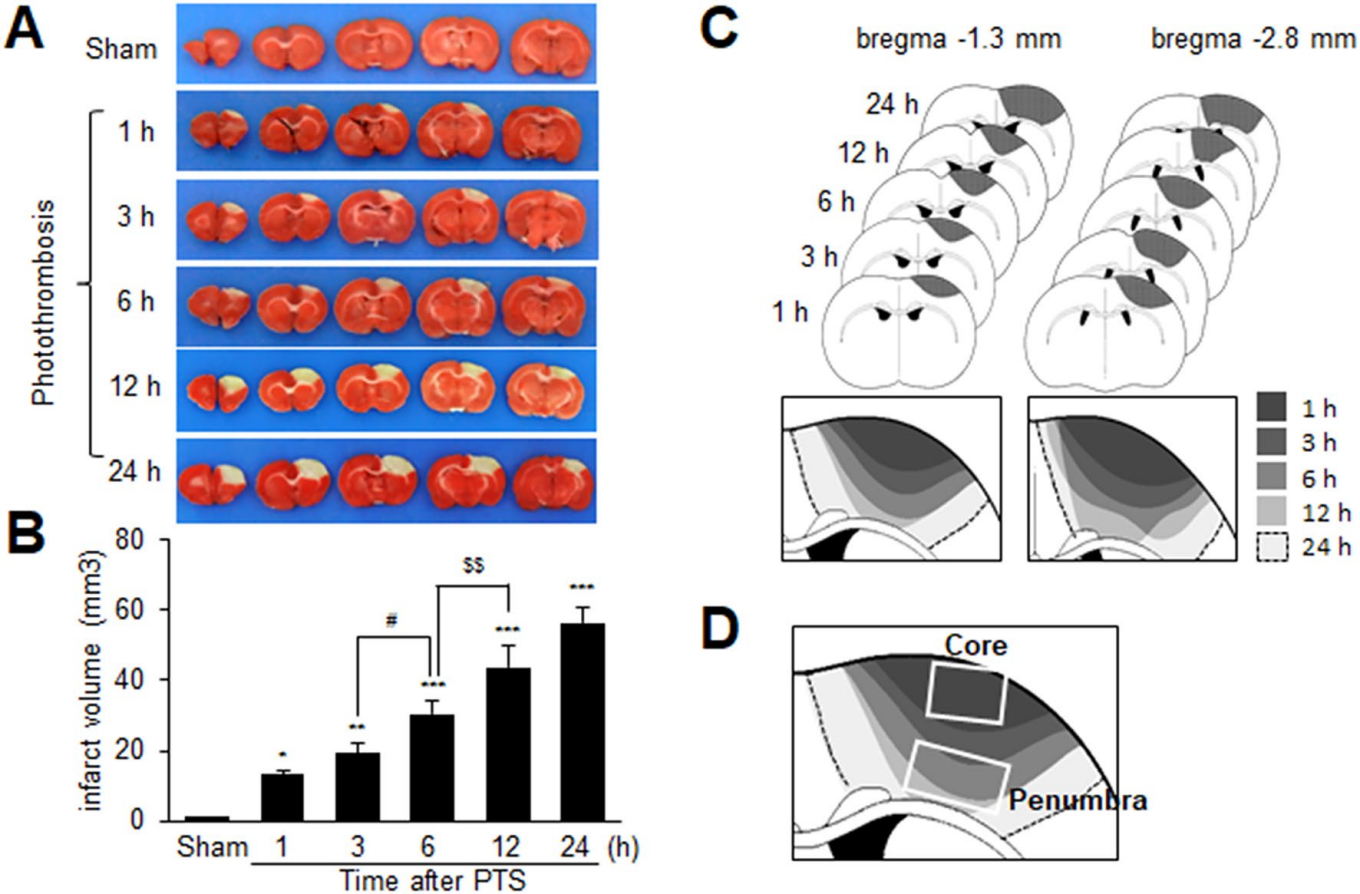
Temporal profile of infarct development in a PTS animal model. **A**, **B** Coronal brain sections were collected at 1, 3, 6, 12, and 24 h after PTS surgery and stained with TTC to measure infarct volumes. **C** Progressive infarct expansion in each brain section obtained 1, 3, 6, 12, or 24 h after PTS was overlaid onto two coronal brain section templates positioned at − 1.3 mm and − 2.8 mm from the bregma. **D** Schematic representation of the brain region depicting the infarct core and penumbra. Data are presented as mean ± SEM (n = 4). *p < 0.05, **p < 0.01, ***p < 0.001 compared to sham-operated groups. ^#^p < 0.05, ^$$^p < 0.01 between indicated groups

To investigate the time course of infarct development during photothrombosis, TTC staining was performed using a PTS model at 1, 3, 6, 12, and 24 h post-surgery. Infarcts were detectable as early as 1 h after surgery, and infarct volumes increased rapidly until 24 h (Fig. 1A, B). These results indicate rapid but progressive infarct expansion in the PTS animal model. The PTS consistently produces cortical lesions with well-defined infarct areas. Reconstruction of the infarct area overlaid on a coronal brain section revealed progressive infarct expansion and localization of the infarct core and the peri-infarct region (penumbra) (Fig. 1C, D).

### Inductions of NETosis (parenchymal and intravascular) in the PTS model

**Fig. 2:**
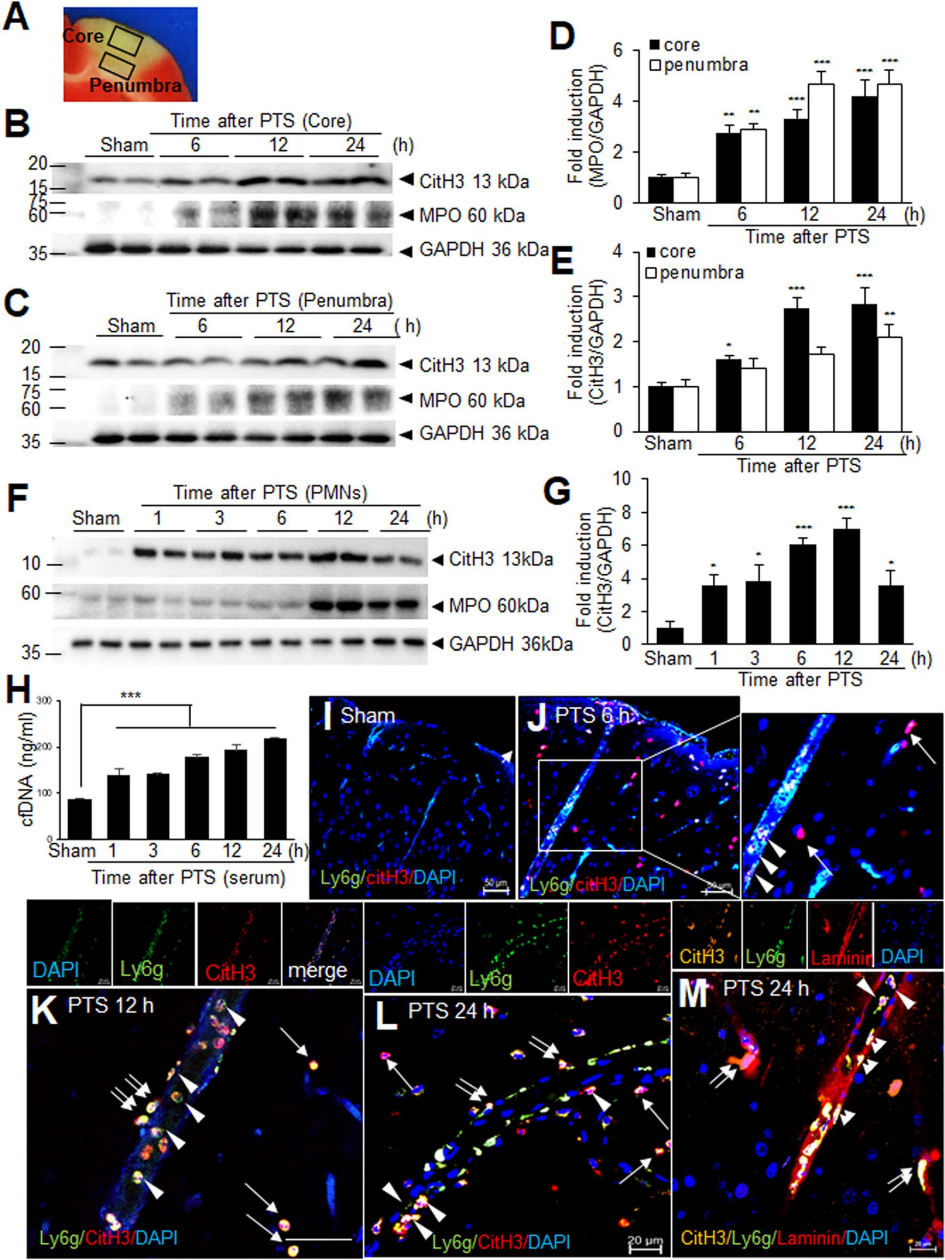
CitH3 induction in brain tissue and PMNs following PTS. **A** Representation of the brain region depicting the infarct core and penumbra following PTS. **B**–**E** Protein levels of MPO (**D**) and CitH3 (**E**) in the cortical core (B) and cortical penumbra (C) 6, 12, and 24 h after PTS induction were evaluated by immunoblotting. **F**, **G** CitH3 and MPO levels were assessed in PMNs isolated 1, 3, 6, 12, and 24 h after PTS induction by immunoblotting. **H** Amounts of cell free DNA in serum were quantified using Quant-iT PicoGreen dsDNA reagent. Representative images are shown in **B**, **C**, and **F** and quantified data are presented as mean ± SEM (n = 4). *p < 0.05, **p < 0.01, ***p < 0.001 compared to sham-operated controls. **I**–**M** Coronal brain sections were prepared from sham controls (**I**) and PTS animals at 6 h (**J**), 12 h (**K**), or 24 h (**L**, **M**) post-PTS. Immunofluorescence staining was performed using anti-Ly6G antibody, anti-CitH3 antibody, and DAPI (**I**–**L**) or with anti-Ly6G antibody, anti-CitH3 antibody, anti-laminin antibody, and DAPI (**M**). Arrows indicate co-localization of anti-CitH3 with anti-Ly6G in non-lytic neutrophils and arrowheads indicate co-localization of anti-CitH3 with anti-Ly6G inside the blood vessels. Double arrows indicate co-localization of anti-CitH3 with anti-Ly6G in extravasating neutrophils and double arrowheads point to localization of anti-CitH3 on the luminal surface of endothelial cells. Scale bars represent 50 µm in **I**–**K**, and 20 µm in **L** and **M**

The observation of rapid lesion growth beyond the illuminated area in the PTS model prompted us to investigation into the potential role of intravascular NETosis in this phenomenon. To elucidate the temporal profile of NETosis induction, we first examined brain tissue at 6, 12, and 24 h after PTS surgery. A significant increase in myeloperoxidase (MPO), a marker of neutrophil activation, was observed at 6 h post-PTS in both the ischemic core and penumbra (Fig. 2A–D). Concomittantly, a significant induction of CitH3, a marker of NETosis, was detected at 6 h post-PTS in the core, with a further pronounced increase at 12 h (Fig. 2B, D). While a slightly delayed induction was observed in the penumbra (Fig. 2B, C, E), these findings collectively demonstrate rapid NETosis induction following PTS. Circulating neutrophils (polymorphic nuclear neutrophils, PMNs) isolated from the peripheral blood of PTS animals exhibited significantly elevated levels of CitH3 and MPO starting at 1 h, further increasing until 12 h and then declining (Fig. 2F, G), strongly suggesting a rapid and prominent induction of intravascular NETosis. Furthermore, serum levels of cell-free DNA, a hallmark of NETosis, were also significantly increased starting at 1 h and continued to increase until 24 h after PTS (Fig. 2H), further supporting this observation. Triple immunofluorescence staining using anti-Ly6G (neutrophil marker) and anti-CitH3 antibodies, along with DAPI, revealed an increase in the number of CitH3-positive cells 6 h after PTS, both in the brain tissue and within blood vessels (arrows and arrowheads, respectively, Fig. 2J). This increase persisted and further intensified at 12 and 24 h post-PTS, particularly within blood vessels (arrowheads) (Fig. 2K, L). Notably, some CitH3^+^/Ly6G^+^ cells appeared to be extravasating from blood vessel (double arrows) (Fig. 2K, L). Importantly, quadruple staining, incorporating an anti-laminin antibody, further confirmed that CitH3^+^/Ly6G^+^ cells exhibited a flattened morphology and adhered to the luminal surfaces of endothelial cell walls (double arrowheads in Fig. 2M). Some of these cells were observed extravasating from the blood vessel (double arrows in Fig. 2M). Collectively, these observations indicate rapid induction of intravascular NETosis following PTS injury.

### Inhibititon of peptidylarginine deiminase 4 (PAD4) suppresses NETosis and infarct formation in the PTS model

**Fig. 3:**
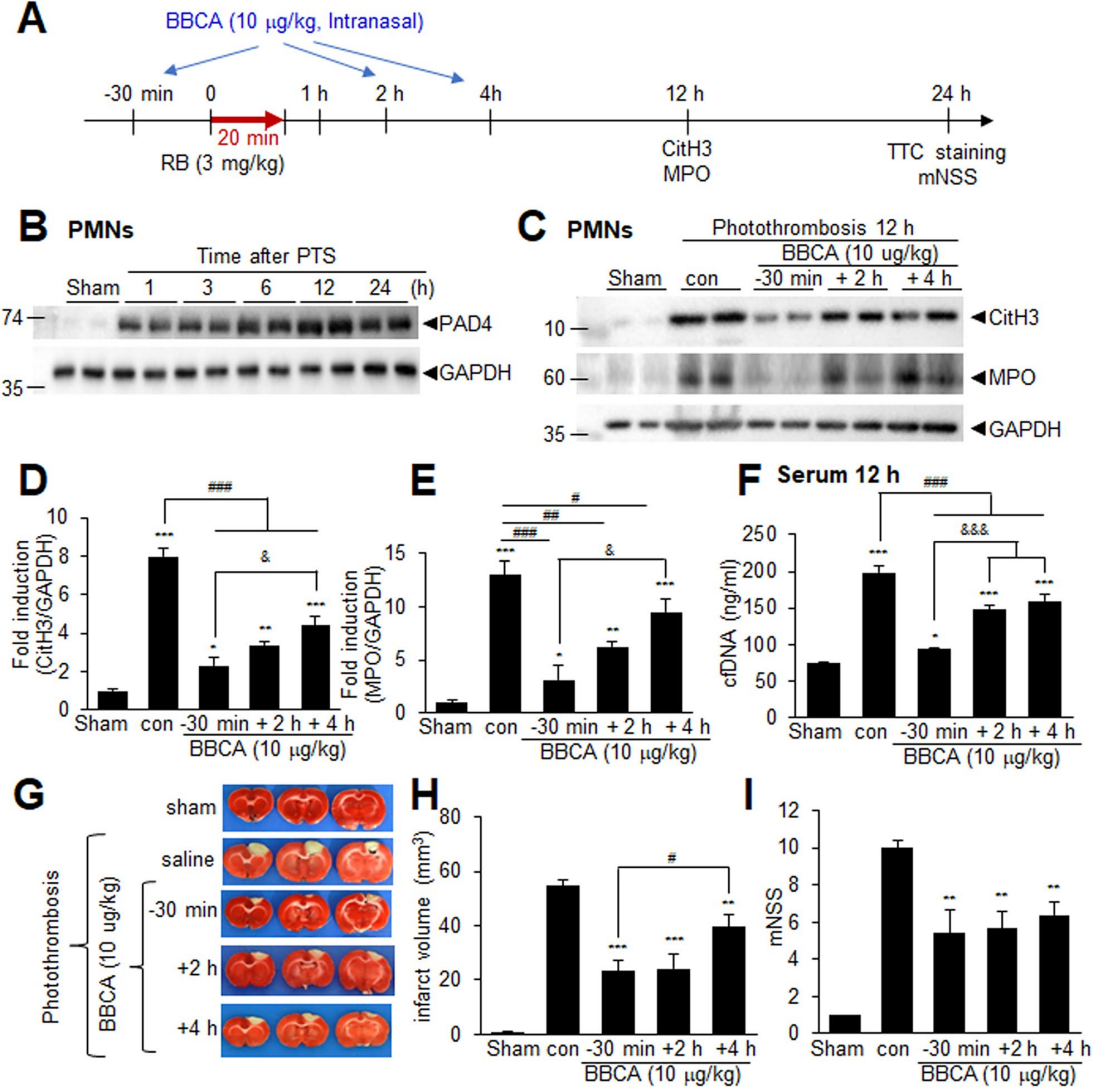
Intranasal treatment of BB-Cl-amidine suppresses NETosis and confers neuroprotection in PTS. **A** BB-Cl-amidine (BBCA, 10 µg/kg) was administered intranasally at 30 min before or 2 or 4 h after PTS induction. **B** Levels of PAD4 were examined in PMNs isolated 1, 3, 6, 12, and 24 h after PTS. **C**–**F** BB-Cl-amidine (BBCA, 10 µg/kg) was administered intranasally at 30 min before or 2 or 4 h after PTS induction. CitH3 and MPO levels were examined in PMNs isolated 12 h after PTS (**C**–**E**). Levels of cell-free DNA in serum were measured 12 h after PTS (**F**). Data are presented as the mean ± SEM (n = 4). Sham, sham-operated animals; PTS, saline-treated PTS control animals; PTS + -30 min, PTS animals administered BBCA 30 min before PTS; PTS + 2 h, PTS animals administered BBCA 2 h after PTS; PTS + 4 h, PTS animals administered BBCA 4 h after PTS. *p < 0.05, **p < 0.01, ***p < 0.001 compared to the sham controls and ^#^p < 0.05, ^##^p < 0.01, ^###^p < 0.001, ^&^p < 0.05, ^&&^p < 0.01 between indicated groups. **G**, **H** Coronal brain sections were obtained at 24 h after PTS and stained with TTC to visualize infarcts. Representative images (**G**) and mean infarction volumes (**H**) are shown. **I** Neurological deficits measured by modified neurological severity scores were evaluated 24 h post-PTS. Data are presented as the mean ± SEM (n = 4). Sham, sham-operated animals (n = 4); MCAO, saline-treated PTS control animals (n = 4); PTS + -30 min, PTS animals administered BBCA 30 min before PTS (n = 4); PTS + 2 h, PTS animals administered BBCA 2 h after PTS (n = 4); PTS + 4 h, PTS animals administered BBCA 4 h after PTS (n = 4). *p < 0.05, **p < 0.01, ***p < 0.001 compared to the sham controls and ^#^p < 0.05 between indicated groups

PAD4 is an enzyme that modifies proteins by converting positively charged arginine residues to neutral citrulline residues (Vossenaar et al. 2003), thereby triggering NETosis through the citrullination of histone H3 (Leshner et al. 2012). PAD4 expression was upregulated in the penumbra but not in the core in the post-PTS brain (Supplementary Fig. 2). However, it was significantly increased in PMNs starting 1 h post-PTS and continued to rise until 12 h (Fig. 3B). To investigate the role of NETosis in infarct formation in the post-PTS brain, we administered BBCA, a well-known NETosis inhibitor that inhibits PAD4, to the PTS animal (Fig. 3A). BBCA (10 µg/kg) was administered intranasally 30 min before or 2 or 4 h after PTS surgery (Fig. 3A). As expected, the induction of CitH3 and MPO observed in PMNs isolated 12 h post-PTS was significantly suppressed by BBCA administration at 30 min before PTS (Fig. 3C–E). Notably, administration of BBCA at 2 or 4 h after PTS also significantly suppressed CitH3 and MPO induction (Fig. 3C–E), indicating that NETosis continued to progress even after the initial ischemic insult. Administration of BBCA 30 min before or 4 h post-MCAO significantly suppressed elevated levels of cell-free DNA in serum at 12 h post-PTS (Fig. 3F), further confirming the effective inhibition of NETosis by post-treatment with BBCA. Importantly, administering BBCA 2 or 4 h after PTS significantly reduced mean infarct volumes at 24 h post-surgery to 44.1 ± 8.7% (n = 4, p < 0.01) or 77.2 ± 5.2% (n = 4, p < 0.01), respectively, compared to saline-treated PTS controls (Fig. 3G, H). Additionally, the mNSS assessed at 24 h post-PTS was also significantly lower in BBCA-treated groups compared to saline-treated PTS controls (10.0 ± 0.6 vs 5.67 ± 0.9 or 6.40 ± 0.7, respectively) (Fig. 3I). These findings demonstrate that NETosis progression occurring after 4 h post-PTS plays a critical role in excerbating damages in the post-PTS brain.

### HMGB1 induction in the brain and peripheral neutrophils following PTS

**Fig. 4:**
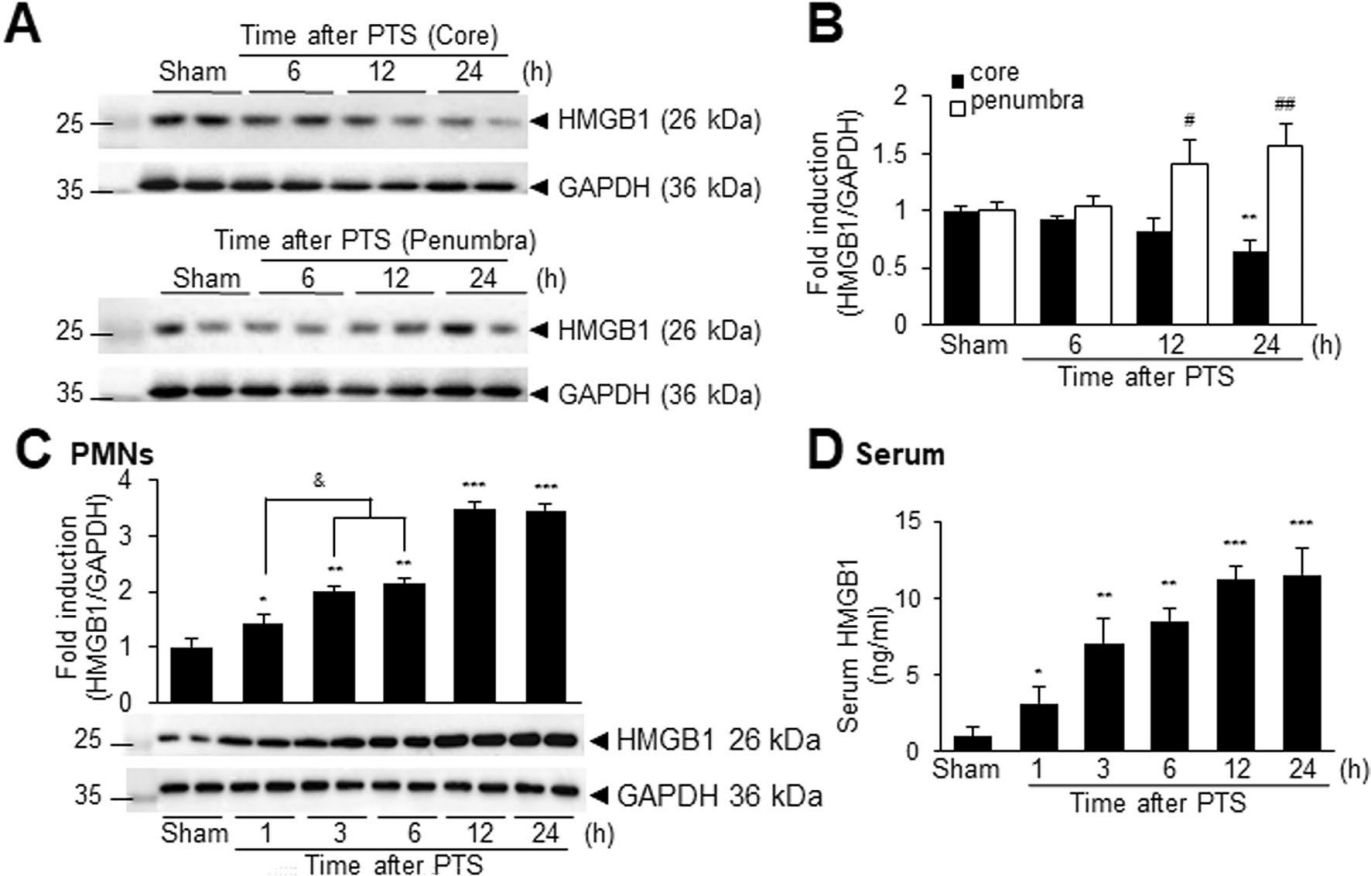
HMGB1 induction in brain tissue, PMNs, and serum after PTS. **A**, **B** HMGB1 levels in the cortical cores or penumbras of ischemic hemispheres were measured 6, 12, and 24 h post-PTS using immunoblotting. **C**, **D** HMGB1 levels in blood PMNs (**C**) and serum (**D**) were assessed 1, 3, 6, 12, and 24 h after PTS using immunoblotting and ELISA, respectively. Results are presented as mean ± SEM (n = 4). *p < 0.05, **p < 0.01, ***p < 0.001 compared to sham controls in the core and PMNs and ^#^p < 0.05, ^##^p < 0.01 compared to sham controls in the penumbra, and ^&^p < 0.05 between indicated groups

Previously, we demonstrated that HMGB1 plays a critical role in inducting NETosis in the brain after permanent MCAO, which was induced using an intraluminal suture method (Kim et al. 2019). To investigate the role of HMGB1 in the PTS model, particularly in neutrophil activation, we examined HMGB1 expression in brain tissue and PMNs and its release into the circulation in this animal model. HMGB1 levels gradually decreased in the infarction core, with a significant reduction observed 24 h post-PTS (Fig. 4A, B). In contrast, HMGB1 expression was slightly but significantly increased in the penumbra region (Fig. 4A, B). PMNs isolated at various time points (1, 3, 6, 12, and 24 h after surgery) exhibited significant HMGB1 induction as early as 1 h, peaking at 12 h (Fig. 4C). Importantly, HMGB1 rapidly accumulated in the serum beginning as early as 1 h post-PTS and continued to rise markedly until 24 h (Fig. 4D). The substantial accumulation of HMGB1 in the bloodstream suggests that HMGB1 induction within the brain parenchyma following PTS might be considerably higher than the levels detected in the immunoblot analysis. This finding underscores the potential significance of HMGB1 in the pathophysiology of cerebral ischemia induced by PTS.

### Localization of HMGB1 not only in neurons but also in activated neutrophils and platelets in the blood vessels following PTS

**Fig. 5:**
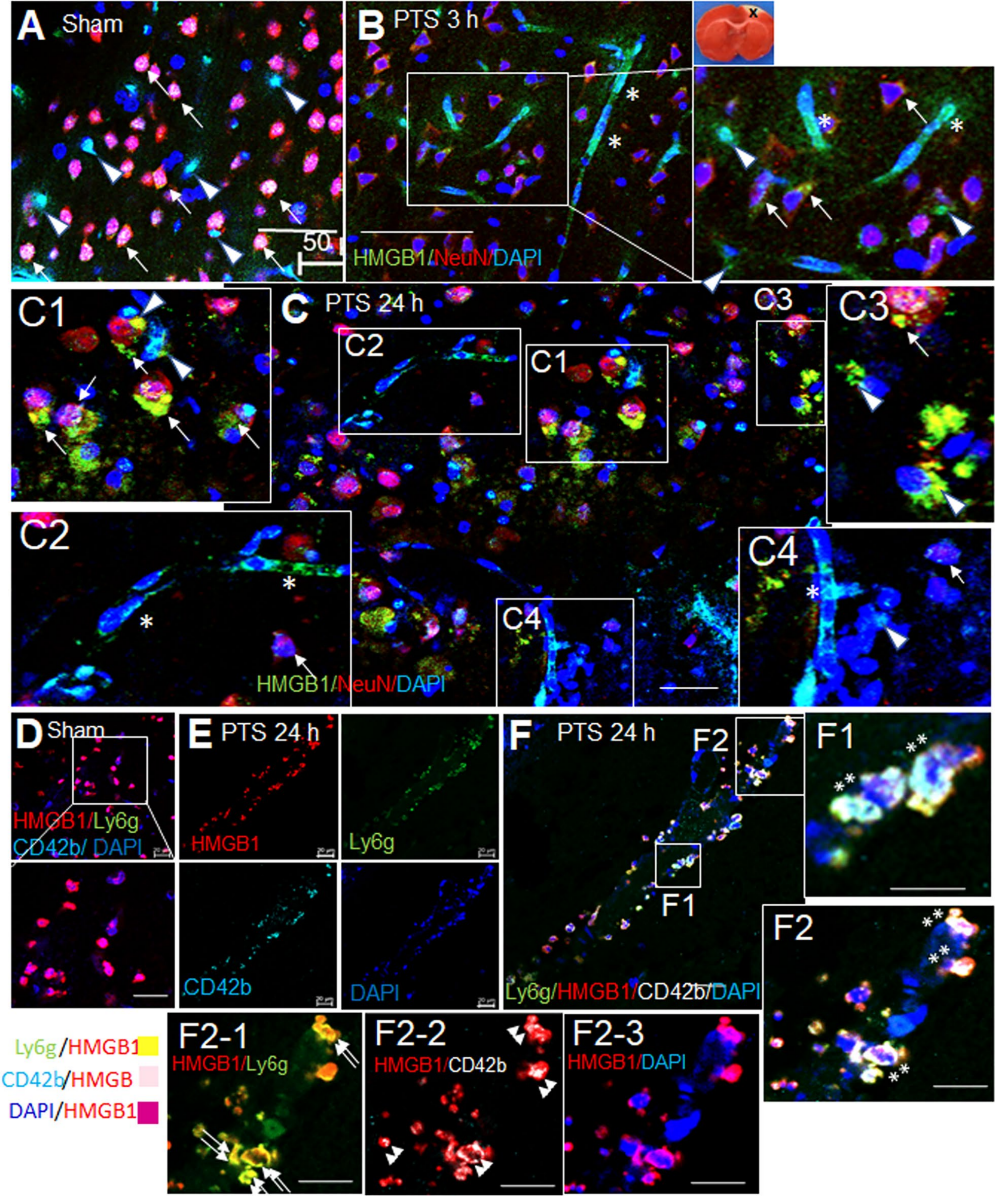
Localization of HMGB1 induction in brain tissue after PTS and its intravascular localization. Coronal brain sections were prepared from sham controls (**A**, **D**) and the penumbra of the ischemic hemisphere of PTS animals at 3 h (**B**) and 24 h post-PTS (**C**, **E**, **F**). Immunofluorescence staining was performed with anti-NeuN antibody, anti-HMGB1 antibody, and DAPI (**A**–**C**) or with anti-Ly6G antibody, anti-HMGB1 antibody, anti-CD42b antibody, and DAPI (**D**–**F**). Arrows indicate co-localization of anti-HMGB1 with anti-NeuN (**B**, **C1**–**C4**), and arrowheads point to localization of anti-HMGB1 in NeuN-negative cells (**A**, **B**, **C1**, **C3**, **C4**), double arrows indicate co-localization of anti-HMGB1 with Ly6G-positive cells (**F2-1**), double arrowheads point to co-localization of anti-HMGB1 in CD42b-positive cells (**F2-2**), and asterisks and double asterisks point to localization of HMGB1 in the blood vessel (**B**, **C2**, **C4**, **F1**, **F2**). Scale bars represent 20 µm in **D**–**F** or 50 µm in **A**, **B**, **C**, **F1**, **F2**, and **F2-1-F2-3**

To examine the cellular localization of HMGB1 in the post-PTS brain, triple immunofluorescence staining was performed using anti-HMGB1 antibody, anti-NeuN antibody (a neuronal marker), and DAPI. In sham controls, HMGB1 immunoreactivity was predominantly observed in the nucleus of neurons (NeuN-positive cells) (arrows, Fig. 5A). HMGB1 immunoreactivity was also detected in NeuN-negative cells, likely microglia or astrocytes, both in the nucleus and cytoplasm (arrowheads, Fig. 5A). At 3 h post-PTS, HMGB1 was detected in the cytoplasm of neurons as scattered puncta (arrows) and in both the nucleus and cytoplasm of NeuN-negative cells (arrowheads) (Fig. 5B). In addition, HMGB1 localization within the blood vessels was notably increased 3 h post-PTS (asterisks, Fig. 5B). At 24 h, HMGB1 immunoreactivity appeared as numerous aggregates in both NeuN-positive and NeuN-negative cells (arrows and arrowheads, respectively, Fig. 5C–C4, with some aggregates seemingly extruded from the cells (Fig. 5C1, C3). Intravascular localization of HMGB1 was abundant, and some appeared to be released from the blood vessels (Fig. 5C, C2, C4). Quadruple immunofluorescence staining using anti-HMGB1 antibody, anti-Ly6g antibody, anti-CD42 antibody (platelets marker) and DAPI (Fig. D-F) showed co-localization of HMGB1 with neutrophils (double arrows, Fig. 5F2-1), platelets (double arrowheads, Fig. 5F2-2), and potentially endothelial cells (Fig. 5F2-3) at 24 h post-surgery (Fig. 5E, F2-3). Notably, HMGB1-positive neutrophils and platelets aggregated in the intravascular space, adhering to the luminal surface of endothelial cells (double asterisks, Fig. 5F, F1, F2). These findings collectively suggest that HMGB1 is induced in PTS model and appears to be co-localized with neutrophils undergoing NETosis and activated platelets.

### A robust neuroprotective effect of HMGB1 A box in part by suppression of intravascular NETosis in the PTS model

**Fig. 6:**
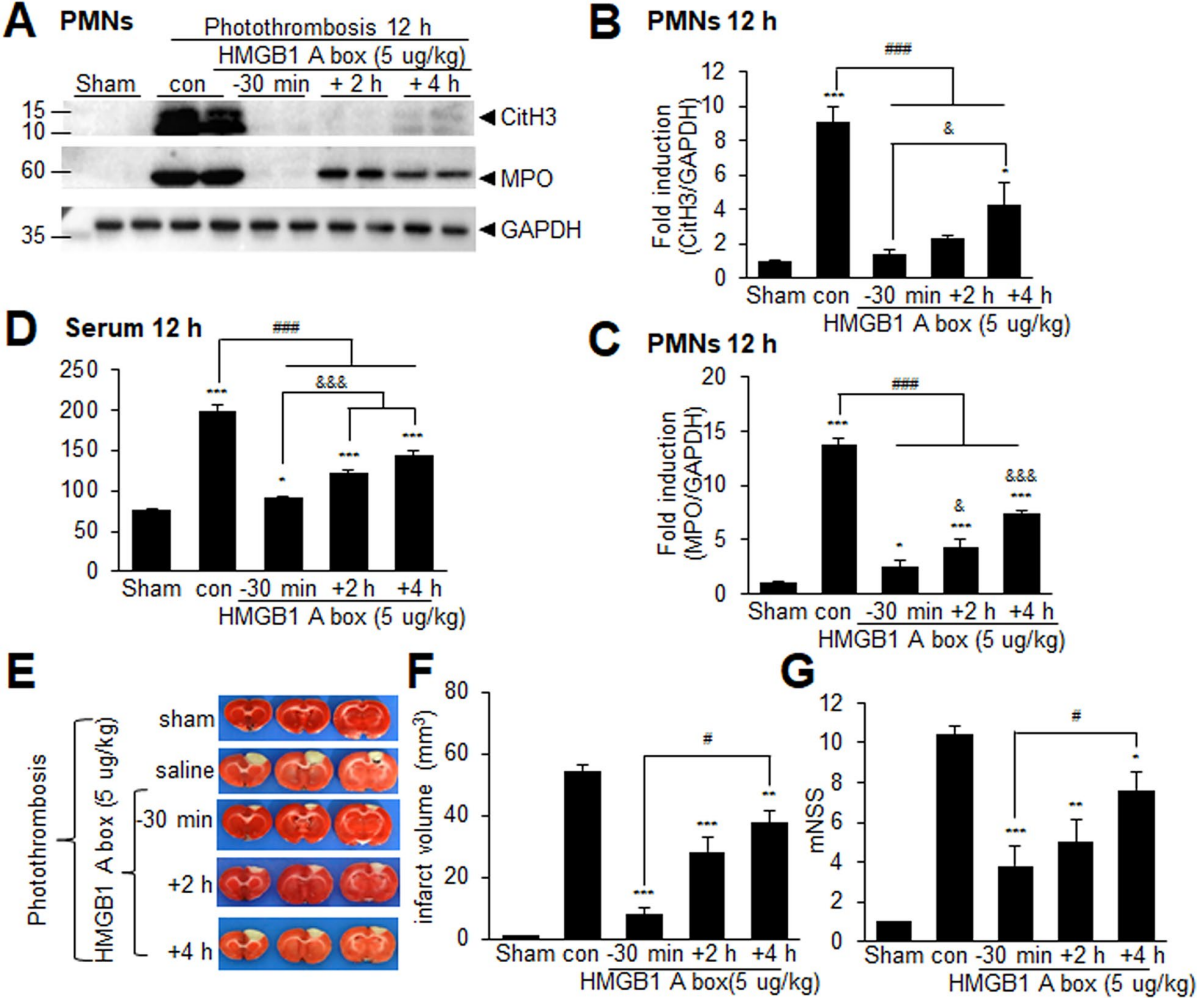
Intranasal administration of HMGB1 A box at 4 h after PTS suppresses NETosis induction in the post-PTS brain. HMGB1 A box (5 µg/kg) was administered intranasally at 30 min before or 2 or 4 h after PTS. **A**–**C** CitH3 and MPO levels were examined in PMNs isolated 12 h after PTS. **D** Levels of cell-free DNA in serum were measured 12 h after PTS. **E**, **F** Coronal brain sections were obtained 24 h after PTS and stained with TTC to visualize the infarcts. Representative images (**E**) and mean infarct volumes (**F**) are shown. **G** Neurological deficits, measured using modified neurological severity scores, were evaluated 24 h post-PTS. Data are presented as the mean ± SEM (n = 4 or 6). Sham, sham-operated animals (n = 4); MCAO, saline-treated PTS control animals (n = 4); PTS + -30 min, PTS animals administered HMGB1 A box 30 min before PTS (n = 6); PTS + 2 h, PTS animals administered HMGB1 A box 2 h after PTS (n = 6); PTS + 4 h, PTS animals administered HMGB1 A box 4 h after PTS (n = 6). *p < 0.05, **p < 0.01, ***p < 0.001 compared to the sham controls and ^#^p < 0.05, ^###^p < 0.001, ^$^p < 0.05 between indicated groups

The substantial accumulation of HMGB1 within blood vessels, along with its co-localization with CitH3, prompted us to investigate whether HMGB1 plays a critical role in NETosis induction following PTS. HMGB1 A box, an antagonistic peptide of HMGB1, was administered intranasally 30 min before, or 2 or 4 h after PTS. Elevated levels of both CitH3 and MPO observed in PMNs at 12 h post-PTS were significantly suppressed in the − 30 min administration groups (Fig. 6A–C). Importantly, significant suppression was also observed with administration at 2 and 4 h post-PTS (Fig. 6A–C). The results demonstrated that intravascular NETosis can be effectively suppressed not only by blocking HMGB1 30 min prior to PTS surgery but also up to 4 h post-surgery. Administration of HMGB1 A box at 4 h post-PTS significantly suppressed the elevated levels of cell-free DNA in serum at 12 h post-PTS (Fig. 6D), further supporting the inhibitory effect of HMGB1 A box on NETosis. We found that intranasal administration of HMGB1 A box (5 µg/kg) at 30 min before, or 2 or 4 h after PTS significantly reduced mean infarct volumes and improved mNSS scores compared to saline-treated PTS controls (Fig. 6E–G). Notably, the suppression of infarct formation achieved with 30 min pretreatment was significantly greater than that observed with 4 h post-administration of HMGB1 A box and 30 min pre-administration of BBCA (Supplementary Fig. 3). Collectively, these findings indicated that HMGB1 inhibition significantly suppressed NETosis induction in PTS animals, resulting in a time-dependent suppression of infarct volume.

### Rapid activation of platelets following PTS

**Fig. 7:**
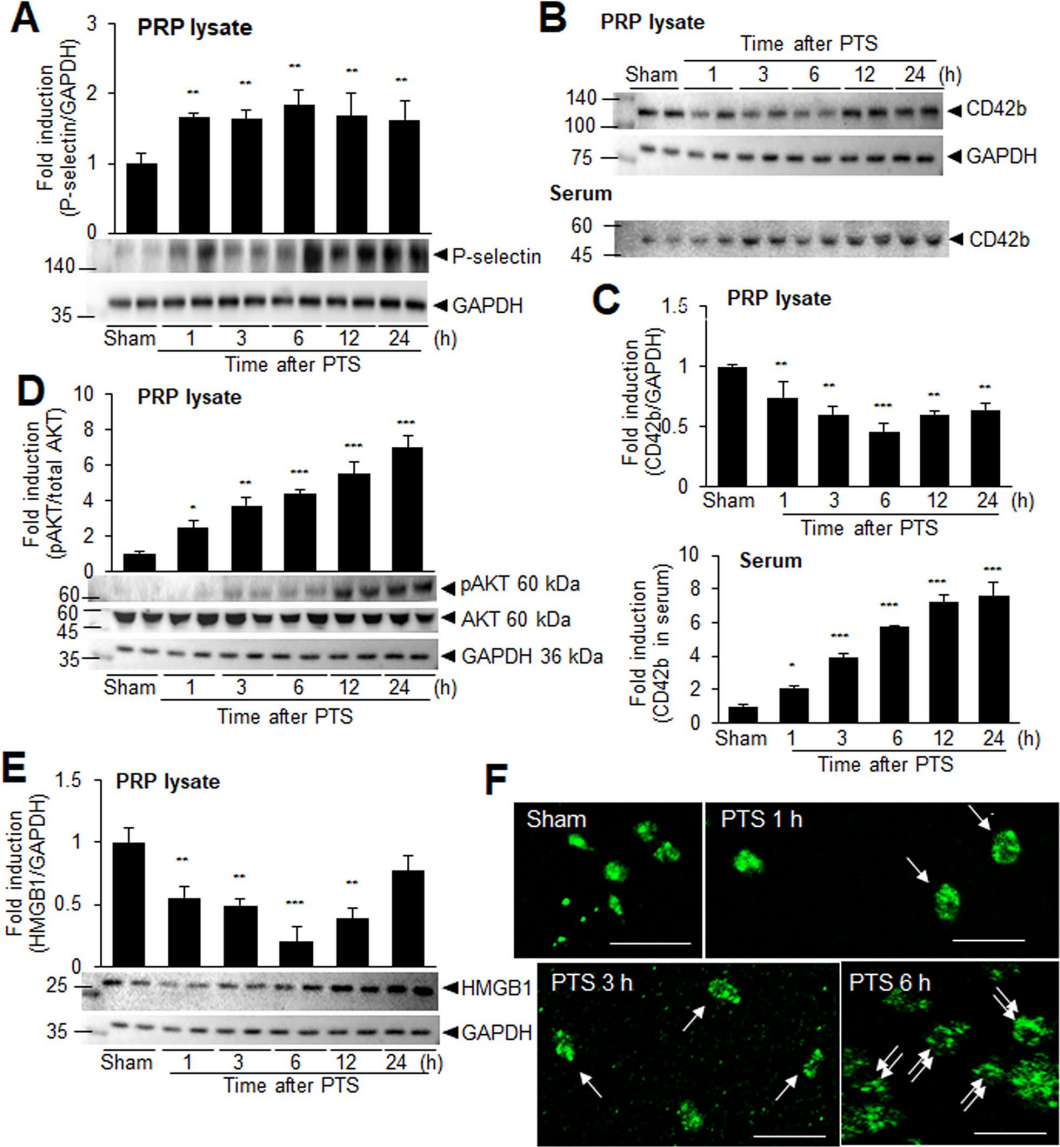
Rapid activation of platelet following PTS. Platelets were isolated 1, 3, 6, 12, and 24 h post-PTS, and protein levels of P-selectin (**A**), CD42b (**B**, **C**), phospho-AKT and AKT (**D**), and HMGB1 (**E**) were examined by immunoblotting. **C** Levels of CD42b were also examined in serum at 1, 3, 6, 12, and 24 h post-PTS by immunoblotting. Representative images are shown and quantified data are presented as mean ± SEM (n = 6 for A, D, E and n = 4 for **C**). *p < 0.05, **p < 0.01, ***p < 0.001 compared to sham group. **F** Platelets were isolated from sham-operated or at 1 and 3 h post-PTS and stained with anti-HMGB1 antibody. Arrows indicate concentrated HMGB1 immunoreactivity in plasma membrane. Double arrows indicate diffused HMGB1 immunoreactivity outer surface of plasma membrane. Scale bars represent 10 µm

To elucidate the role of HMGB1, particularly platelet-derived HMGB1, in intravascular NETosis induction following PTS, we first examined the temporal profiles of platelet activation. Plate-rich plasma (PRP) was isolated at various time points (1, 3, 6, 12, and 24 h) after PTS surgery. The levels of P-selectin (CD62P), a marker of platelet activation, were significantly elevated in the PRP as early as 1 h post-PTS, and the enhanced levels were maintained until 24 h (Fig. 7A). GP1bα (CD42b) levels, another platelet activation marker, showed a significant decrease in platelets isolated 1 h after PTS, followed by a gradual decrease. Conversely, GP1bα levels in serum gradually and significantly increased (Fig. 7B, C). This observation suggests ectodomain shedding of CD42b from the platelet surface, a phenomenon reported to occur through ADAM 17-mediated proteolysis in activated platelets (Six et al. 2023). In addition, the levels of phosphorylated-AKT (p-AKT), a downstream molecule of PI3K, which is involved in platelet activation, were significantly increased in platelets isolated 1 h after PTS and continued to rise (Fig. 7D). Together these results indicate rapid activation of platelets following PTS. Importantly, HMGB1 levels in platelets decreased markedly 1 h after PTS and further decreased until 6 h and then gradually returned to baseline (Fig. 7E). This likely reflects translocation of HMGB1 to the plasma membrane and subsequent secretion, as reported by Vogel et al. (2015). We also observed a rapid translocation of HMGB1 immunoreactivity to the plasma membrane within 1–3 h following activation (arrows, Fig. 7F). Subsequently, the immunoreactivity diffused and localized to the outer surface of platelets (double arrows, Fig. 7F). Collectively, all these findings indicate rapid activation of platelets following PTS, accompanied by the secretion of HMGB1 from activated platelets.

### Critical role of platelet HMGB1 in neutrophil activation via TLR4 signaling in PTS

**Fig. 8:**
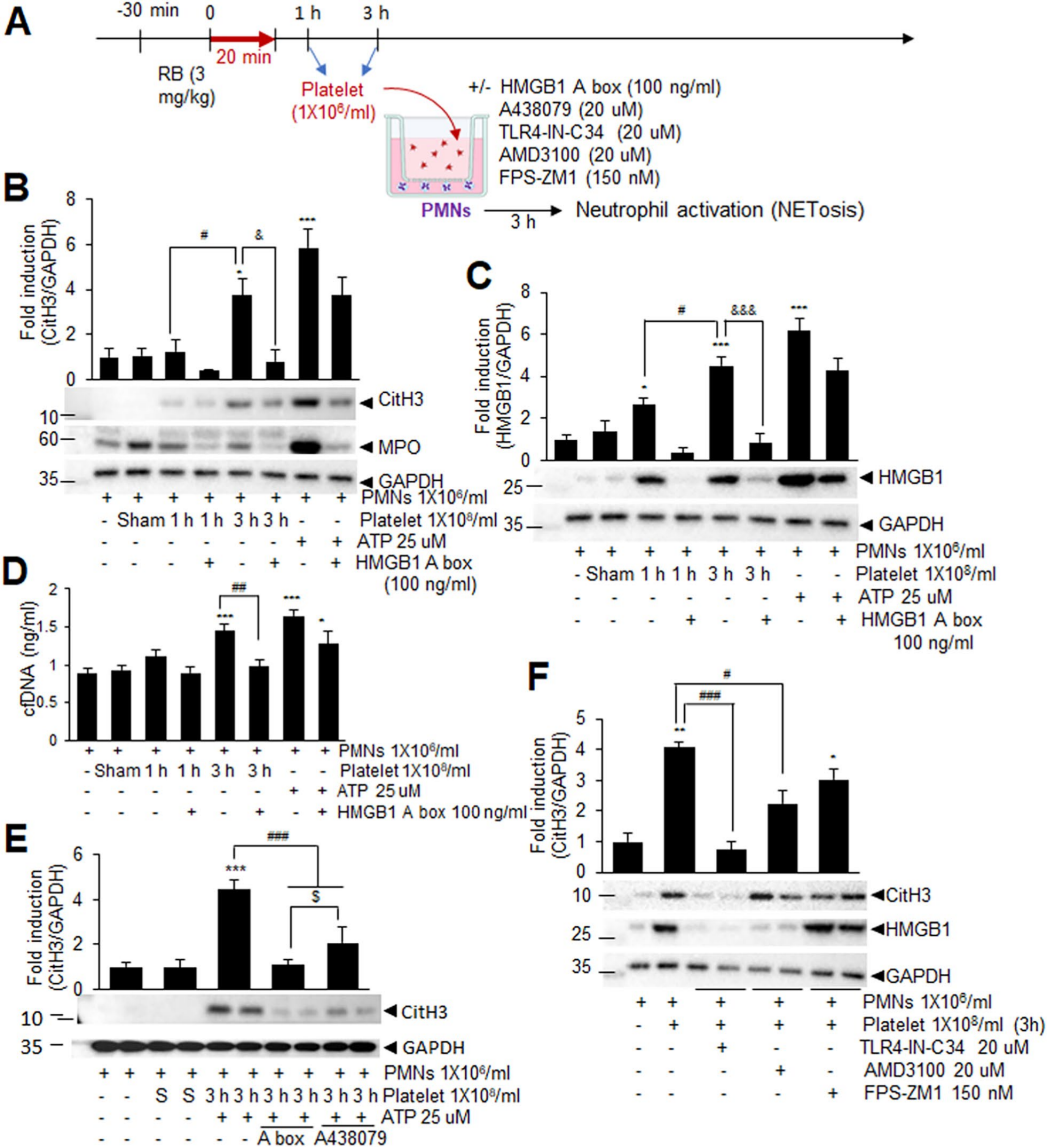
Platelet HMGB1 triggers NETosis following PTS in TLR4- and CXCR4-dependent manner. **A** Schematic illustration of the co-culture protocol for normal PMNs and platelets isolated from PTS animals. **B**–**E** Normal PMNs were seeded onto six-well-plate and cultured for 24 h and then co-cultured with platelets isolated from sham-operated or at 1 or 3 h post-PTS animals for 3 h. (**B**–**E**) PMNs were pre-incubated with HMGB1 A box (100 ng/mL) or A438079 (20 µM) for 30 min before co-culture with platelets. ATP treatment (25 µM) was used as a positive control. Protein expression of CitH3, MPO, and HMGB1 was assessed by immunoblotting (**B**, **C**, **E**) and levels of cell-free DNA in serum were measured (**D**). **F** PMNs were pre-incubated with TLR4-IN-C34 (20 µM), AMD3100 (20 µM), or FPS-ZM1 (150 nM) for 30 min before co-culture with platelets. Representative images are shown and quantified data are presented as mean ± SEM (n = 4–6). *p < 0.05, ***p < 0.001 compared to sham group and ^#^p < 0.05, ^##^p < 0.01, ^###^p < 0.001, ^&^p < 0.05, ^&&&^p < 0.001, ^$^p < 0.05 between indicated groups

To investigate whether HMGB1 derived from activated platelets following PTS can induce NETosis, co-culture experiments were conducted with normal (naïve) PMNs and platelets isolated from sham-operated or 1 or 3 h post-PTS (Fig. 8A). Almost no increase in CitH3 and MPO levels were observed in PMNs after 3 h of co-culture with platelets isolated from sham-operated and 1 h post-PTS animals (Fig. 8B). However, CitH3 and MPO levels were significantly elevated when PMNs were co-cultured with platelets isolated 3 h post-PTS (Fig. 8B). Importantly, these NETosis marker inductions were significantly attenuated when PMNs were pre-incubated with HMGB1 A box for 30 min prior to co-culture with platelets (Fig. 8B). Notably, levels of HMGB1 were significantly induced in PMNs treated with platelets isolated 1 or 3 h post-PTS, and this increase was suppressed by the HMGB1 A box (Fig. 8C). These results indicate that platelets isolated 3 h post-PTS are capable of inducing NETosis, with HMGB1 playing a crucial role in this process. Furthermore, levels of cell-free DNA in the serum were significantly increased by co-culturing with platelets isolated at 3 h post-PTS and decreased back to basal level by the HMGB1 A box (Fig. 8D), further supporting the role of platelet-derived HMGB1 in NETosis induction. ATP served as a positive control, with 25 µM ATP significantly inducing NETosis (Fig. 8B), as previously reported (Kim et al. 2020). Interestingly, pre-treatment of PMNs with A438079, an antagonist of P2X7 receptor (P2X7R) significantly suppressed CitH3 induction, although to a lesser extent than the HMGB1 A box (Fig. 8E), suggesting a potential role of ATP in platelet-mediated NETosis induction. Furthermore, the NETosis-inducing effect was significantly suppressed when PMNs were co-incubated with platelets together with TLR4-IN-C34 (a TLR4 inhibitor). However, the suppressive effect was weaker with AMD3100 (a CXCR4 inhibitor), and not significant with FPS-ZM1 (a RAGE antagonist, 150 nM) (Fig. 8F). Collectively, these findings suggest that platelet-derived HMGB1 plays a critical role in neutrophil activation in the PTS animal model, and that TLR4 signaling appears to play an important role in this process.

## Discussion

In the PTS model, thrombus formation occurred immediately after RB-laser treatment (within 10 min), as evidenced by a drastic reduction in cerebral blood flow measured by laser Doppler flowmetry (Pena-Martinez et al. 2019) (Supplementary Fig. 1). Moreover, the decreased blood flow remained constant, indicating that the resulting clot was stable (Pena-Martinez et al. 2019). Based on this observations, the PTS model can be considered as a model of permanent ischemia. We previously demonstrated the critical role of HMGB1 in permanent MCAO generated by the intraluminal suture method (Kim et al. 2019). The present study provides evidence that HMGB1 plays a critical role in the pathogenesis of PTS by mediating the interaction between neutrophils and platelets.

In the PTS model, thrombi are fibrin-free and primarily composed of aggregated platelets and neutrophils (Watson et al. 1985; Dietrich et al. 1986; Uzdensky 2018). The critical role of NETosis in thrombus formation in PTS models has been reported in several studies. Pena-Martinez et al. (2019) demonstrated that pretreatment with Cl-amidine, a PAD4 inhibitor, 10 min prior to photothrombotic insult, prevented thrombotic occlusion by inhibiting NETosis. Additionally, NET formation was suppressed in the PAD4-deficient photothrombotic MCAO model (Wang et al. 2021). Moreover, administration of DNase-I at 3 h post-ischemic insult recanalized the occluded blood vessels in the PTS animal model (Pena-Martinez et al. 2019; Wang et al. 2021). While these reports indicate NET formation during the initial thrombus formation induced by Rose Bengal and irradiation, the present study demonstrated that NETosis is a progressive phenomenon. Specifically, intravascular NETosis continued to increase, contributing to the exacerbation of brain damage following PTS. Significant suppression of NETosis along with robust neuroprotective effects of BBCA administered 2 or 4 h post-PTS confirmed that delayed NETosis plays a critical role in exacerbating brain damage after PTS. However, it is important to acknowledge that, in addition to NETosis, protein modifications mediated by PAD4-catalyzed citrullination (deamination) may also contribute to the detrimental consequences in the post-PTS brain. This possibility is supported by numerous reports reveling PAD4-mediated citrullination in various neuropathological conditions, such as multiple sclerosis (Sarswat et al. 2017), Alzheimer’s disease (Ishigami et al. 2005), and cerebral ischemia (Seol et al. 2024). Notably, significant PAD4 upregulation was observed not only in PMNs but also in various cell types within the brain tissue following PTS (Fig. 3B; Supplementary Fig. 2), further supporting this possibility. However, future investigation is warranted to fully elucidate the specific role of PAD4-mediated citrullination in the context of post-PTS brain damage.

Following PTS, intravascular neutrophil activation and NETosis induction occurred rapidly, commencing within 1 h after the insult, and persisting up to 12 h. Of note, a portion of the CitH3-positive neutrophils detected within the blood vessels exhibited lytic features, adhering to endothelial cells or extravasating from the vessels. NETs release numerous cytotoxic substances, including neutrophil elastase, MPO, histones, and HMGB1, which directly or indirectly damage endothelial cells, leading to increased vascular permeability (Kim et al. 2019; Zenaro et al. 2015; Santos-Lima et al. 2022; Saffarzadeh et al. 2012). Recently, El Amki et al. (2020) reported that neutrophils adhering to distal capillary segments caused microvascular obstruction in both the core and penumbra regions of a thrombin-induced stroke model, and the removal of circulating neutrophils using an anti-Ly6G antibody restored microvascular perfusion. The formation of microthrombi by aggregated neutrophils has also been reported to induce occlusion in small vessels adjacent to the infarcted area in the distal MCA model (Erdener et al. 2021), and the importance of NETs in the microthrombus phenomenon has been demonstrated (Thalin et al. 2016). Therefore, intravascular NETosis, particularly involving lytic neutrophils accumulating within the blood vessels in the post-PTS brain, likely plays crucial roles not only in the initial thrombus formation but also in the gradual expansion of the infarct following PTS.

**Fig. 9:**
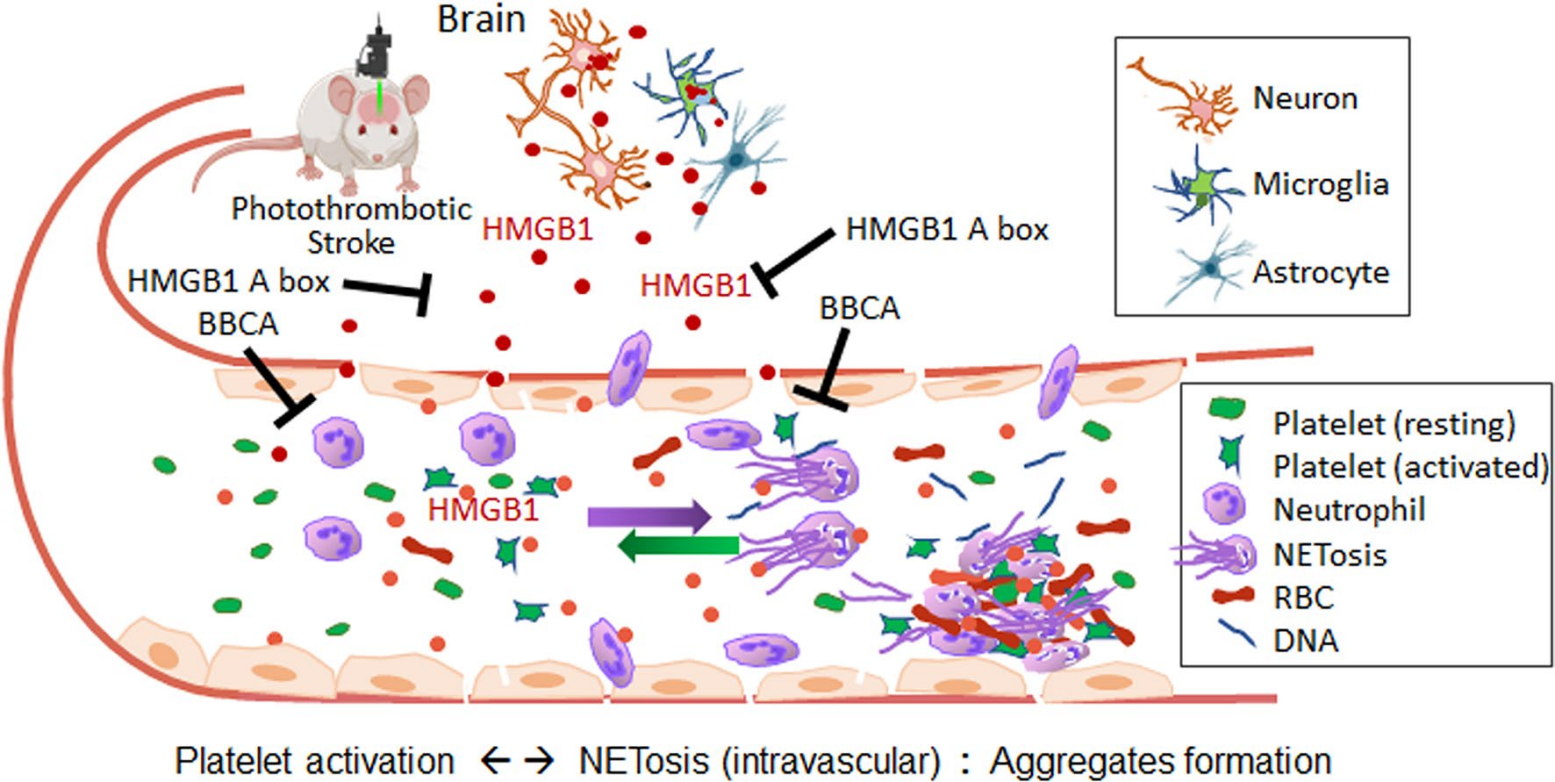
Diagram of the interplay between activated platelets and neutrophils in PTS and the function of HMGB1. HMGB1 is released from neurons and glia during the acute phase after PTS. This released HMGB1 accumulates within blood vessels, activating platelets and inducing NETosis. Activated platelets release HMGB1, further amplifying NETosis. Platelets adhere to the endothelium and interact with NETs, trapping circulating procoagulant factors and red blood cells (RBCs). This leads to the formation of neutrophils-platelets aggregates, where HMGB1 derived from both activated platelets and NETosed neutrophils likely plays a critical role. Administration of BBCA or HMGB1 A box can suppress this process. HMGB1, High mobility group box 1; BBCA, BB-Cl-amidine; RBCs, red blood cells

The kinetics of HMGB1 induction and extracellular release following PTS exhibited distinct characteristics compared to those after both transient and permanent MCAO (Kim et al. 2006, 2012, 2019). Specifically, a rapid and prominent induction of HMGB1 was observed in PMNs, with only a moderate increase in the penumbra and a significant decrease in the core. Given the rapid and robust surge in serum HMGB1 levels (Fig. 4D), it is evident that a substantial amount of HMGB1 is released extracellularly following PTS, likely originating from various sources, including damaged neurons, activated glia, endothelial cells, and NETosed neutrophils. Although, serum HMGB1 may originate from different cellular sources, co-culture experiments have demonstrated a critical role for platelet-derived HMGB1. While a critical role for platelet-derived HMGB1 has been established in coronary thrombi (Maugeri et al. 2014), experimental trauma/hemorrhagic shock (Vogel et al. 2015), and venous thrombosis (Stark et al. 2016), the present study confirms its significance in the PTS model. Notably, the present study revealed prominent and rapid induction of HMGB1 not only in platelets but also in PMNs following PTS. Considering that HMGB1 is secreted (extruded) from neutrophils as a component of NETs (Kim et al. 2019) and that HMGB1-induced platelet activation is well-documented (Vogel et al. 2015; Nolfi-Donegan et al. 2024), HMGB1 likely plays bidirectional roles in the interplay between neutrophils and platelets in the post-PTS brain. Therefore, along with those HMGB1 derived from multiple sources as mentioned above, platelet-derived HMGB1 likely serves as a crucial mediator in a vicious cycle that exacerbates brain damage following PTS (Fig. 9).

To the best of our knowledge, this is the first report characterizing the temporal dynamics of HMGB1 induction and its cellular and subcellular localization following PTS. Nuclear to cytoplasmic translocation of HMGB1 was observed in both neurons (NeuN-positive cells) and non-neuronal cells (NeuN-negative cells), with prominent cytoplasmic accumulation forming large aggregates that appeared to be extruded from the cells. This observation likely reflect the extensive neuronal necrosis occurring during the acute phase after PTS, with the released HMGB1 contributing significantly to the observed serum HMGB1 surge. Subsequently, HMGB1 was detected within aggregates of activated platelets and neutrophils in blood vessels. While a major criticism of the PTS model is the lack of a salvageable penumbra, a significant reduction in infarct volume achieved by intranasal administration of HMGB1 A box at 4 h post-PTS suggest a presence of viable penumbra that may be a target for neuroprotective interventions. It is important to point out that pre-administration of HMGB1 A box demonstrated a markedly greater infarct suppressive effect compared to post-administration. Given that HMGB1 is well-known to exert potent neuroprotective effects in the post-ischemic brain, attributed to its anti-excitotoxicity (Kim et al. 2011), anti-inflammatory (Kim et al. 2018), anti-NETosis (Kim et al. 2006, 2019; Qiu et al. 2008), and anti-ferroptotic properties (Davaanyam et al. 2023, 2024), earlier inhibition of HMGB1 may offer protection from various perspectives. In support of this, we demonstrated significant suppression of proinflammatory cytokine induction in the post-PTS brain following HMGB1 A box administration (Supplementary Fig. 4). Considering that NETosis has been implicated in the exacerbation of inflammation, particularly in cerebral ischemia (Denorme et al. 2022; Vogel et al. 2015), HMGB1 might be directly or indirectly involved in inflammation in the post-PTS brains.

Two distinct redox forms of HMGB1 exist: disulfide type HMGB1 (dsHMGB1) and reduced type HMGB1 (reHMGB1), each exerting distinct functions in various pathological contexts. DsHMGB1 activates the TLR4/MD-2 complex, functioning as a cytokine (Tadie et al. 2013) and reHMGB1 recruits neutrophils and monocytes to inflammatory sites by forming a complex with CXCR12 and binding to CXCR4 (Venereau et al. 2012). Regarding NETosis induction, we previously reported that both types of HMGB1 can induce NETosis following permanent MCAO, albeit with differential kinetics (Kim et al. 2019). In the present study, platelet-derived HMGB1-induced NETosis was more effectively suppressed by a TLR4 inhibitor, suggesting a critical role for the dsHMGB1-TLR4 signaling pathway. This result is in line with previous reports demonstrating the importance of TLR4 in HMGB1-mediated NETosis induction in animal models of inflammatory liver injury (Huang et al. 2015), acute lung injury (Zhan et al. 2022), and thromboembolic stroke (Pena-Martinez et al. 2022). However, the precise potencies and differential functions of different HMGB1 redox forms have not been fully investigated. Therefore, further research is necessary to elucidate the specific contributions of different HMGB1 redox forms to neutrophils-platelets interaction and to identify the involved receptors.

The present study demonstrated that NETosis exacerbate inflammation and contributed to subsequent brain damage following PTS. Inhibition of NETosis achieved by BBCA or HMGB1 A box significantly reduced infarct volume and improved neurological outcomes in an animal model of PTS. Platelets-derived HMGB1 plays a crucial role in inducing NETosis through a TLR4-dependent mechanism. These results suggest NETosis serve as a valuable prognostic marker in acute ischemic stroke. Furthermore, targeting NETosis through a modulation of HMGB1 may offer a promising therapeutic strategy for mitigating ischemic brain damage.

## Supplementary Information

Supplementary Material 1.

## Data Availability

No datasets were generated or analysed during the current study.
