# Supplementary material for: Platelet-derived HMGB1 induces NETosis, exacerbating brain damage in the photothrombotic stroke model
Source: Mol Med. 2025 Feb 5;31:46. doi: 10.1186/s10020-025-01107-7 (PMC11796003; doi:10.1186/s10020-025-01107-7)
Supplement: Supplementary file 1 — Supplementary Material 1. [file 10020_2025_1107_MOESM1_ESM.docx]

**Supplementary materials**

**Platelet-derived HMGB1 induces NETosis, exacerbating brain damage in the photothrombotic stroke model**

Sang-A Oh^1^, Song-I Seol^1^, Dashdulam Davaanyam^1^, Seung-Woo Kim^2^, Ja-Kyeong Lee^1*^

^1^Department of Anatomy, Inha University School of Medicine, ^2^Department of Biomedical sciences, Inha University School of Medicine, Inchon, Republic of Korea

Key words: HMGB1, NETosis, Photothrombosis, platelet, TLR4

Running title: HMGB1 induces NETosis in the photothrombotic stroke model

^*^ Corresponding author:

Ja-Kyeong Lee, Ph.D.

Department of Anatomy, Inha University School of Medicine. iinha 100, Nam-Gu, Inchon, 22212, Republic of Korea, Tel, +82-32-860-9893; FAX, +82-32-884-2105; [jklee@inha.ac.kr](mailto:jklee@inha.ac.kr)

**Materials and Methods**

**C**

**A**

**Reverse transcription‑quantitative polymerase chain reaction (RT‑qPCR)**

Total RNA was extracted from infarct regions of rat brain tissues using the TRIzol reagent (Invitrogen, Carlsbad, CA, USA). cDNA was synthesized using the iScript cDNA Synthesis Kit (Bio-RAD, Hercules, CA, USA) and quantitative real-time PCR (RT-qPCR) was performed. Reactions were conducted using TOPreal qPCR 2X PreMIX SYBR Green with low ROX (Enzynomics, Daejeon, Korea) with the following cycles: initial denaturation at 95 °C for 15 minutes, followed by 55 cycles of denaturation at 95 °C for 10 seconds and annealing/extension at 55 °C for 15 seconds. Primer sequences for the target genes are listed. All PCR reactions were performed in triplicate, and the average threshold cycle (Ct) values were calculated for each sample. Gene expression levels were normalized to the housekeeping gene GAPDH and relative gene expression levels were calculated using the Livak (2 − ΔΔCt) method.

| **Gene**  **(GenBank Accession No.)** | **Oligonucleotide Primer Sequences** | **PCR Product Size (bp)** |
| --- | --- | --- |
| IL-1β  (NM_008361.4) | 5’-GGA GAA GCT GTG GCA GCT A -3’  5’-GCT GAT GTA CCA GTT GGG GA -3’ | 271 |
| TNF-α  (NM_013693.3) | 5’- CAC CAC GCT CTT CTG TCT ACT -3’  5’- GTA CTT GGG CAG ATT GAC CTC -3’ | 422 |
| iNOS  (NM_001313921.1) | 5’- GCA TCC CAA GTA CGA GTG GT -3’  5’- CCA TGA TGG TCA CAT TCT GC -3’ | 252 |
| GAPDH  (NM_001411841.1) | 5’- TCA TTG ACC TCA ACT ACA TGG -3’  5’- CTA AGC AGT TGG TGG TGC AG -3’ | 363 |

**Supplementary figure 1**

**
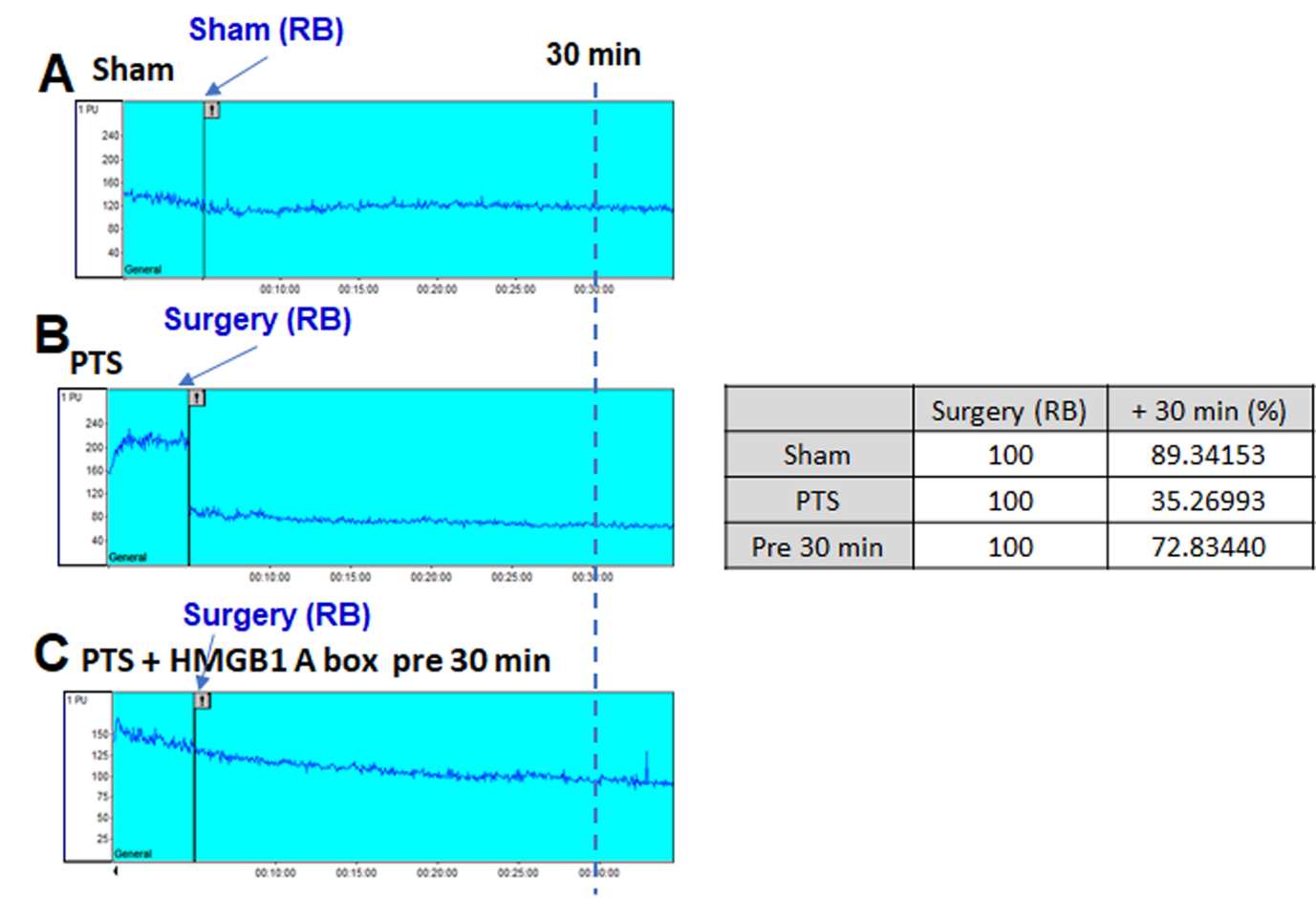
**

**Supplementary figure 1. Doppler flowmeter**

Laser Doppler flowmetry changes through PTS procedure. The baseline blood flow is considered to be 100% for all animals. Change in LDF after occlusion of PTS is demonstrated. Sham, sham-operated animals; PTS, saline-treated PTS control animals; PTS + HMGB1 A box pre 30 min, PTS animals administered HMGB1 A box 30 min before PTS.

**Supplementary figure 2**

**C**

**A**


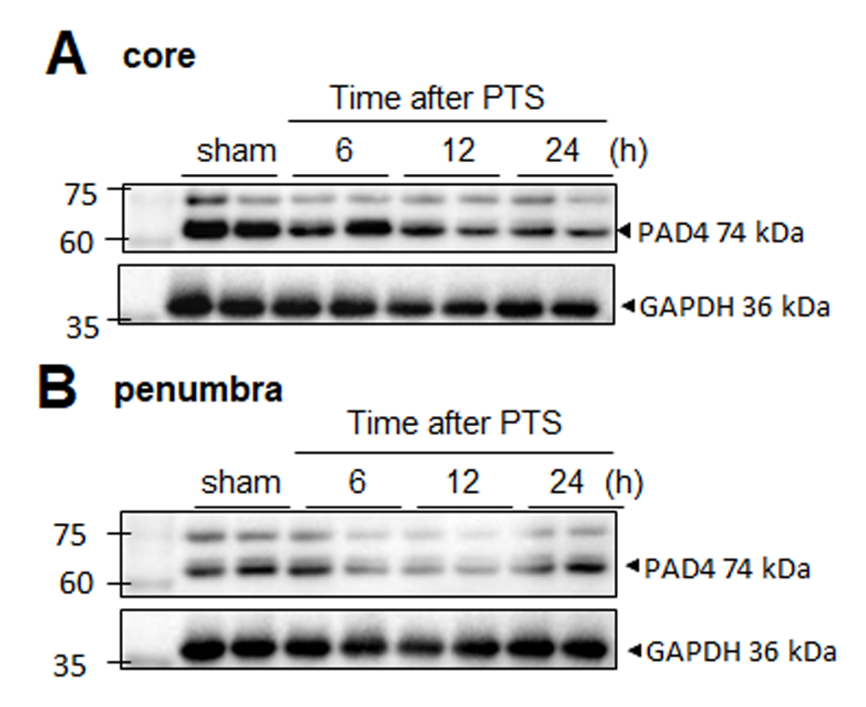


**Supplementary figure 2. PAD4 induction in brain tissue following PTS**

Protein levels of PAD4 in the cortical core (A) and cortical penumbra (B) at 6, 12, and 24 h after PTS induction were evaluated by immunoblotting.

**Supplementary figure 3**

**C**

**A**


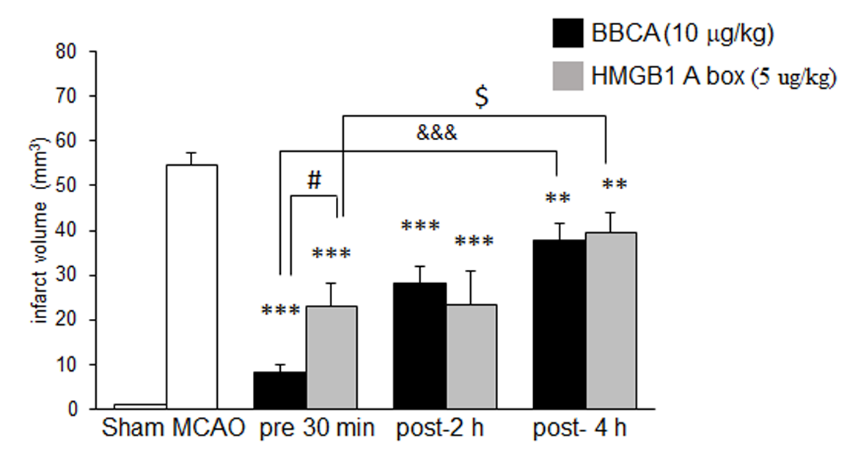


**Supplementary figure 3. Suppression of infarct volumes by pre- or post-administration of BBCA and HMGB1 A box in PTS**

BBCA (10 mg/kg) or HMGB1 A box (5 ug/kg) was administered intranasally either 30 min before or 2 or 4 h after PTS. Coronal brain sections were obtained 24 h after PTS and stained with TTC to visualize the infarcts. Mean infarct volumes measured at 24 h post-PTS are presented as the mean ± SEM. Sham, sham-operated animals; MCAO, saline-treated PTS control animals; PTS + -30 min, PTS animals administered BBCA or HMGB1 A box 30 min before PTS; PTS + post-2 h, PTS animals administered BBCA or HMGB1 A box 2 h after PTS; PTS + 4 h, PTS animals administered BBCA or HMGB1 A box 4 h after PTS. **p < 0.01, ***p < 0.001 compared to the sham controls and ^#^p < 0.05, ^&&&^p < 0.001, ^$^p < 0.05 between indicated groups.

**Supplementary figure 4**

**C**

**A**

**
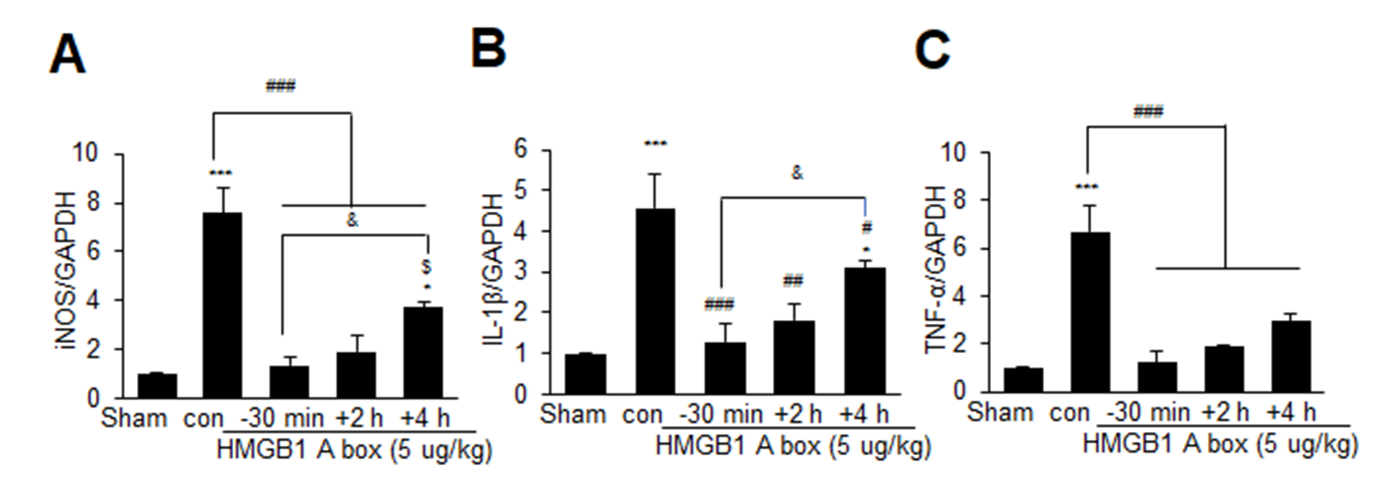
**

**Supplementary figure 4. Anti-inflammatory effects of pre- or post-administration of HMGB1 A box in PTS**

HMGB1 A box (5 µg/kg) was administered intranasally immediately 30 min before or 2 or 4 h after PTS. Levels of iNOS, IL-1β, and TNFα in penumbras of ischemic hemispheres at 24 h post-PTS were determined by RT-qPCR. Results are presented as means ± SEMs (n=4). Sham, sham-operated animals; MCAO, saline-treated PTS control animals; PTS + -30 min, PTS animals administered HMGB1 A box 30 min before PTS; PTS + 2 h, PTS animals administered HMGB1 A box 2 h after PTS; PTS + 4 h, PTS animals administered HMGB1 A box 4 h after PTS . *p < 0.05, ***p < 0.001 compared to the sham controls and ^#^p < 0.05, ^##^p < 0.01, ^###^p < 0.001, ^&^p < 0.05, ^$^p < 0.05 between indicated groups.
